# Efficacy of Acetylshikonin in Preventing Obesity and Hepatic Steatosis in db/db Mice

**DOI:** 10.3390/molecules21080976

**Published:** 2016-07-28

**Authors:** Mei-Ling Su, Yu He, Qi-Sen Li, Bang-Hao Zhu

**Affiliations:** Department of Pharmacology, Cardiac and Cerebral Vascular Research Center, Zhongshan School of Medicine, Sun Yat-sen University, No. 74 Zhongshan Rd. 2, Guangzhou 510080, China; sumeiling124@163.com (M.-L.S.); 13760686022@163.com (Y.H.); XY_Sen@hotmail.com (Q.-S.L.)

**Keywords:** acetylshikonin, obesity, nonalcoholic fatty liver disease, lipid metabolism, inflammatory cytokines

## Abstract

Zicao (*Lithospermum erythrorhizon*) has been used in clinics as a traditional Chinese medicine for thousands of years. Acetylshikonin (AS) is the main ingredient of Zicao, Xinjiang, China. The objective of this study was to investigate the anti-obesity and anti-nonalcoholic fatty liver disease (NAFLD) efficacy of AS in a model of spontaneous obese db/db mice. Mice were divided into Wild Type (WT) groups and db/db groups, which received no treatment or treatment with 100 mg/kg/day clenbuterol (CL) hydrochloride or 540 mg/kg/day AS by oral gavage for eight weeks. The results provided the evidence that AS prevented obesity and NAFLD including reduction in body weight, food efficiency ratio, serum triglyceride (TG) and free fatty acid (FFA) levels in db/db mice. Administration of AS markedly suppressed the levels of hepatic alanine aminotransferase (ALT), aspartate aminotransferase (AST) and pro-inflammatory cytokines in treated groups when compared with that of db/db groups. Further investigation of the lipid synthesis-related protein using Western blotting revealed that hepatic protein expression of sterol regulatory element-binding protein-1 (SREBP-1), fatty acid synthetase (FAS) and 3-hydroxy-3-methylglutaryl-coenzyme A reductase (HMGCR) were significantly downregulated by AS treatment. These findings suggest that AS exerts anti-obesity and anti-NAFLD effects through the regulation of lipid metabolism and anti-inflammatory effects.

## 1. Introduction

Obesity is an increasing risk factor of various metabolic diseases, including type 2 diabetes mellitus (T2DM), insulin resistance, hyperlipidemia, and NAFLD [1]. Obesity-related NAFLD is a clear risk factor of death in the general population around the world. NAFLD has typical characteristics with ectopic fat accumulation in hepatocytes. The ectopic fat accumulation can impair hepatic function, developing into nonalcoholic steatohepatitis (NASH), cirrhosis, and liver cancer [2,3]. Estimates of NAFLD prevalence were approximately 20%–25% worldwide; and the prevalence incidence of NAFLD was much higher in people with obesity than in normal populations [4].

At the first stage of NAFLD, fatty liver is characterized by ectopic fat droplet accumulation in hepatocytes and meanwhile is associated with elevation of liver enzyme levels such as ALT and AST, which is reversible. Hormone-sensitive lipase (HSL), adipose TG lipase (ATGL), and perilipin are crucial enzymes of lipolysis that catalyze the intracellular triacylglycerol to release glycerol and non-esterified fatty acid [5,6]. Lipid metabolism contains lipolysis and lipid synthesis. SREBP-1 plays an essential role in modulating the expression of lipid synthesis-related enzymes [7]. Studies have been reported that SREBP-1 played an important role in the fat droplet infiltration of fatty liver, indicating the expression of SREBP-1 was significantly increased in NAFLD [8].

In the second stage of NAFLD, fatty liver turns into a steatohepatitis, which is accompanied by inflammatory reaction [9]. Evidence manifests that obesity is closely associated with the development of inflammatory in liver diseases including NAFLD and NASH [10]. Although the NAFLD progression is complex and remains enigmatic, several pro-inflammatory cytokines are important regulatory factors in NAFLD. Various pro-inflammatory cytokines, such as tumour necrosis factor-a (TNF-α), interleukin-6 (IL-6) and interleukin-1β (IL-1β) contribute to steatohepatitis development and progression [11,12,13]. Therefore, it is a promising target in the prevention and treatment of NAFLD through inhibiting pro-inflammatory cytokines production and reducing inflammatory reaction.

**Figure 1:**
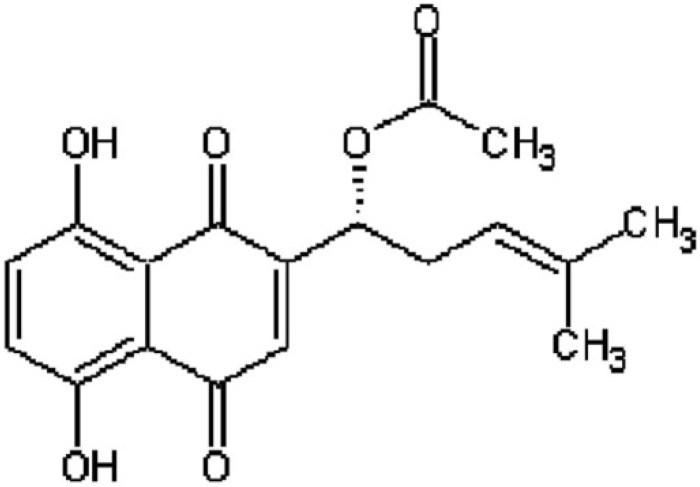
Chemical structures of acetylshikonin.

Although studies have revealed that some pharmacological agents exert beneficial effects on rat models of NAFLD, none have shown promise for reversing NAFLD so far. Therefore, the research and development of an efficient anti-NAFLD drug is urgent and desired. Zicao has been used in traditional herbal medicine for thousands years in China, and its primary active ingredients are naphthoquinone derivatives containing AS (Figure 1), shikonin, β-hydroxyisovaleryshikonin, deoxyshikonin, β, β-dimethylacrylshikonin and isovalerylalkannin [14]. Its derivatives have revealed various pharmacological activities including anti-tumorigenic, anti-bacterial, anti-viral, anti-inflammatory, and wound healing bioactivity [15,16]. Zicao was obtained from Xinjiang in China and AS is the main ingredient of Zicao. In addition, previous study has reported that arnebin-1 (naphthoquinone derivative of Zicao) accelerated the wound healing process in diabetic rats. Moreover, our previous studies have demonstrated that AS could prevent obesity in rats on a high-fat diet model [17]. These suggest that AS will become a beneficial and valuable agent. The present study aimed to investigate the effect of it in preventing or treating obesity and NAFLD in a model of db/db mice, and further clarify its underlying molecular mechanisms.

## 2. Results

### 2.1. AS Suppressed Obesity in db/db Mice

**Figure 2:**
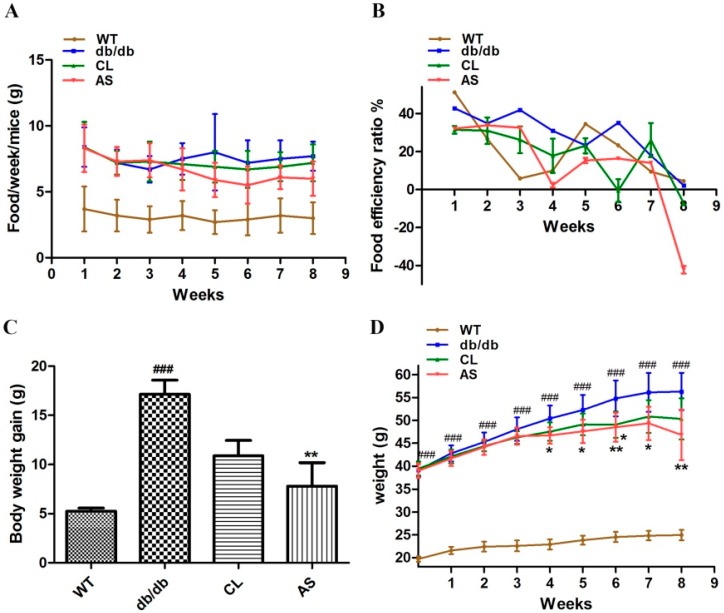
AS suppressed weight gain in db/db mice. Mice were divided into four groups: WT control; obese db/db control; CL treatment (100 mg/kg/day) and AS treatment (540 mg/kg/day). The drugs were oral gavage for eight weeks. (**A**) food intake was recorded weekly; (**B**) food efficiency ratio was calculated as body weight gain divided by food intake; (**C**) change in body weight was measured weekly; and (**D**) body weight gain was measured weekly. Values are expressed as mean ± SEM (*n* = 24). * *p* < 0.05, ** *p* < 0.01 vs. db/db group; ^###^ *p* < 0.001 vs. WT group.

Eight-week-old male C57BL/6J mice were divided into four groups: WT control; obese db/db control; CL treatment (100 mg/kg/day) and AS treatment (540 mg/kg/day). The food intake and body weight of the mice were measured weekly during the experimental period. As shown in Figure 2A, weekly food intake per mouse was not different among all groups during the experimental period (*p* > 0.05). Eight-week administration of CL and AS suppressed the average weight gain by 36.6% and 54.4% vs. db/db group *p* < 0.05, respectively (Figure 2C,D). Moreover, the food efficiency ratio was significantly decreased in CL and AS treated groups by 33.7% and 48.2% when compared with that of obese db/db group (Figure 2B). There were not any pathological signs in the mice during the experimental period. These results demonstrate that both CL and AS efficiently reduce the weight gain in db/db mice.

### 2.2. Effect of AS on Abdominal Adiposity in db/db Mice

**Figure 3:**
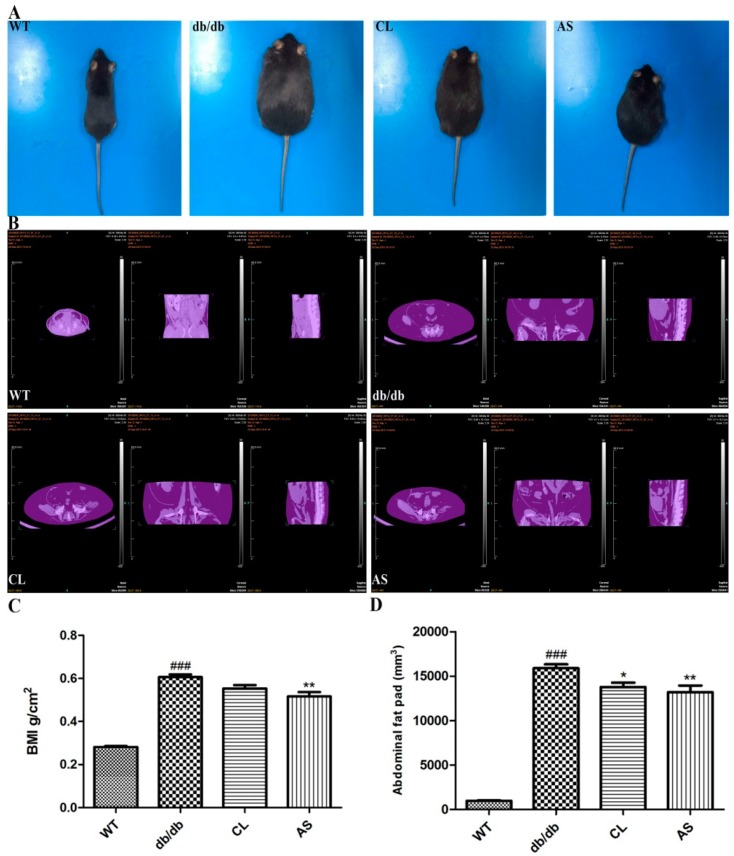
Effect of AS on abdominal adiposity in db/db mice. (**A**) mice images were acquired by a digital camera at the end of experiment; (**B**) micro-computed tomography (CT) images of abdominal fat of mice in WT, db/db, CL, or AS group; (**C**) BMI = weight (g)/length/length (cm^2^); (**D**) fat volumes (mm^3^) in mice are shown. The values represent the mean ± SEM (*n* = 6). * *p* < 0.05, ** *p* < 0.01 vs. db/db group; ^###^ *p* < 0.001 vs. WT group.

Mice images were acquired by a digital camera at the end of the experiment (Figure 3A). For the determination of abdominal adiposity, we performed micro-CT imaging to assess the effect of CL and AS; Figure 3B showed that body fat was reduced by CL and AS treatment (WT group, 941.08 mm^3^; db/db group, 15,896.82 mm^3^; CL group, 13,452.87 mm^3^, AS group, 13,184.78 mm^3^; respectively). In addition, the body mass index (BMI) of obese db/db mice was 2.2-fold higher than that of the WT group, while it was decreased by 15.4% and 17.1% in db/db mice with CL and AS treated groups, when compared with obese db/db group (*p* < 0.05; *n* = 6; Figure 3C). Fat volume in obese db/db mice was 16.3-fold higher than that of the WT group. It was significantly reduced by 8.8% and 14.9% in CL and AS treated groups when compared with the obese db/db group (*p* < 0.05; *n* = 6; Figure 3D).

### 2.3. AS Reduced Adipose Tissue, Liver Weight and Hepatic Lipid Accumulation in db/db Mice

**Figure 4:**
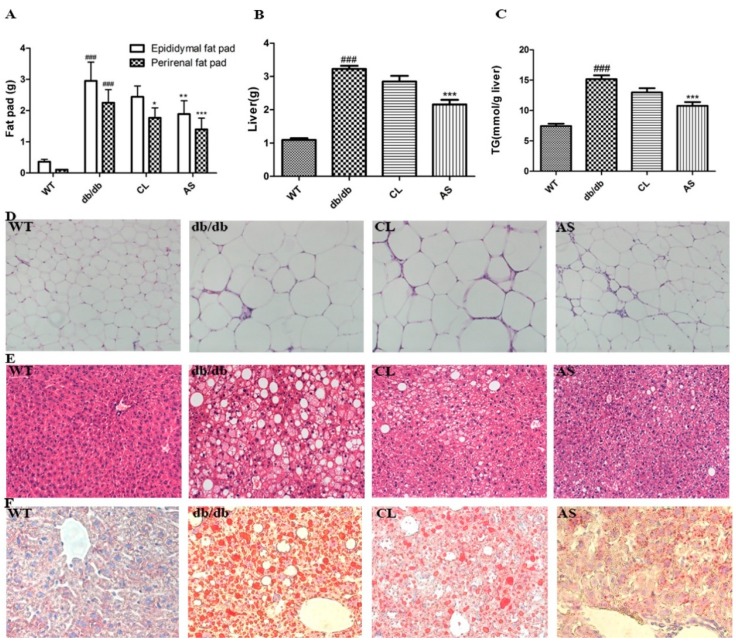
AS reduced adipose tissue, liver weight and hepatic lipid accumulation in db/db mice. (**A**) epididymal and perirenal fat pads; (**B**) liver weight; (**C**) hepatic TG was measured after mice had fasted for 12 h at the end of experiment; (**D**) epididymal adipose tissue and (**E**) liver were stained with hematoxylin and eosin; and (**F**) liver was stained with Oil Red O to detect the quantities of lipids. Images were acquired at 200× magnification by a microscopy. Values are expressed as mean ± SEM (*n* = 6). * *p* < 0.05, ** *p* < 0.01, *** *p* < 0.001 vs. db/db group; ^###^ *p* < 0.001 vs. WT group.

Administration of CL and AS markedly suppressed the increase in the amount of epididymal, perirenal fat pads, and liver weight (*p* < 0.05; Figure 4A,B). The hepatic TG level of db/db mice was 2.03-fold higher than that of the WT group, while it was markedly decreased by 14.4% and 28.9% in CL and AS treated groups when compared with the obese db/db group (*p* < 0.05; *n* = 6; Figure 4C). The pathological signs, including morphological and histological changes in the adipose tissue and liver, were ameliorated in the CL and AS treated groups (Figure 4D,E). Furthermore, the deposition of lipid droplets in the hepatocytes was significantly inhibited in the CL and AS treated groups (Figure 4F) as detected by Oil Red O staining. These results suggest that AS reduces adipose tissue mass and prevents fat accumulation in the liver of db/db mice.

### 2.4. AS Decreased the Blood Lipids and Hepatic Enzyme Levels in db/db Mice

Table 1 shows that the plasma biochemical levels of TG, FFA, CHE, AST, ALT, and glucose were significantly increased in obese db/db group compared with that of the WT group. However, plasma TG and FFA levels were decreased by 56.8% and 46.0% in db/db mice treated with AS, respectively, relative to obese db/db group (*p* < 0.01; *n* = 6). Similarly, the plasma FFA level was reduced by 40.2% in db/db mice treated with CL, as compared with obese db/db group (*p* < 0.01). Meanwhile, the plasma glucose, CHE, AST and ALT levels of db/db mice were 4.9-, 2.0-, 1.6- and 4.5-fold higher than that of WT group, respectively. Administration of AS plasma glucose, CHE, AST and ALT levels were notably decreased by 34.1%, 45.5%, 27.2% and 45.8% in db/db mice, respectively, relative to obese db/db group (*p* < 0.05; *n* = 6). These results show that obesity and NAFLD are ameliorated in the blood lipids and hepatic enzyme levels by AS treatment.

### 2.5. AS Increased the Expressions of Lipid-Metabolizing Enzymes in Adipose Tissue

**Figure 5:**
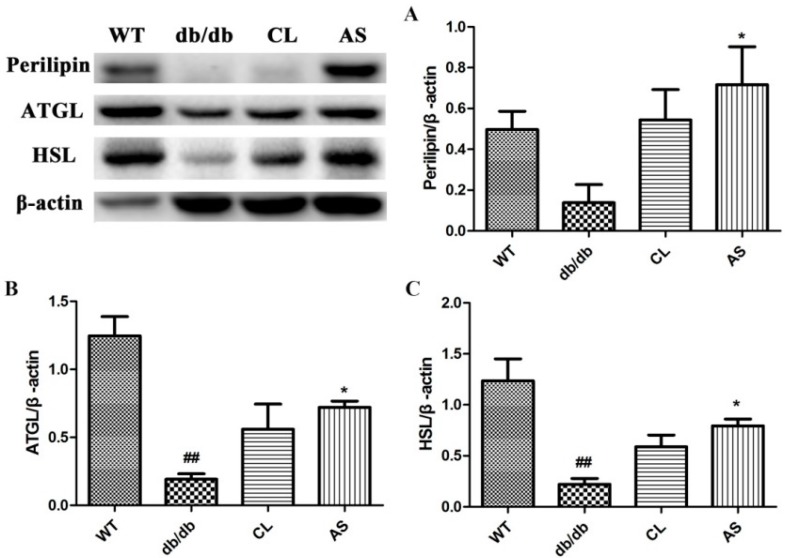
AS increased the expressions of lipid-metabolizing enzymes in adipose tissue. (**A**) perilipin; (**B**) ATGL; and (**C**) HSL protein expression in adipose tissue was analyzed by Western blot. Immunoblots are representative of five independent experiments; β-actin served as a loading control. Values are expressed as mean ± SEM of five mice. * *p* < 0.05 vs. the db/db group; ^##^ *p* < 0.01 vs. the WT group.

Perilipin, ATGL and HSL protein levels were markedly reduced in the obese db/db group compared with that of the WT group; however, they were increased by 3.93-, 2.91-, and 2.66-fold in the CL treated group when compared with the obese db/db group, respectively. In the case of the AS group, they were increased by 5.18-, 3.74-, and 3.58-fold, respectively. (*p* < 0.05; *n* = 5; Figure 5). These data indicate that AS increases key lipid-metabolizing enzymes in adipose tissue.

### 2.6. AS Inhibited the Lipid Synthesis-Related Protein Expressions (SREBP-1, FAS and HMGCR) in the Liver

**Figure 6:**
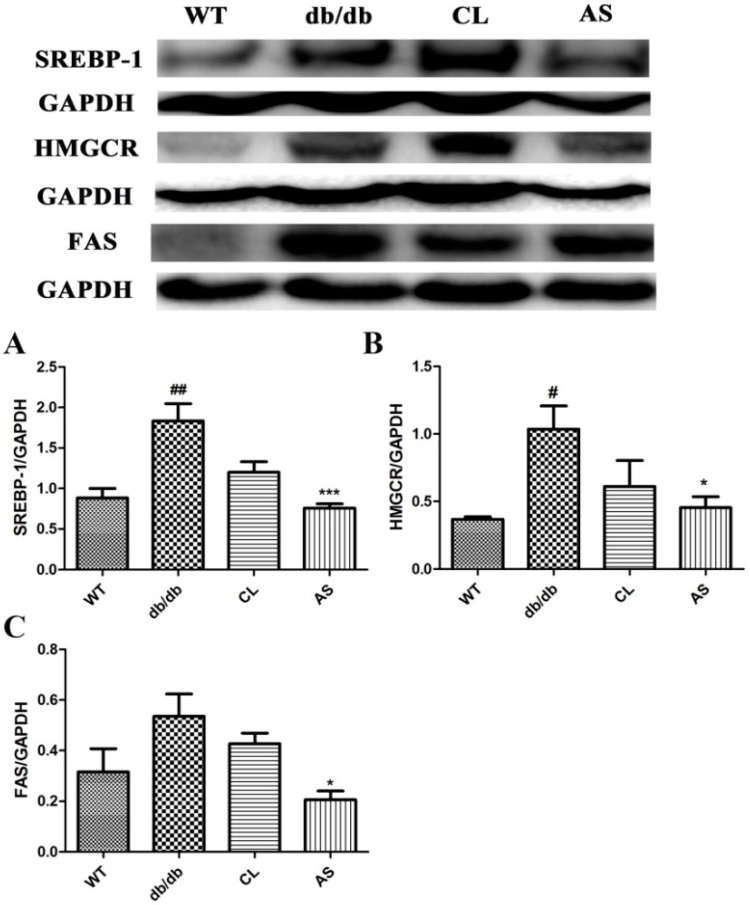
AS inhibited the lipid synthesis-related protein expressions in liver. (**A**) SREBP-1 and (**B**) HMGCR; (**C**) FAS protein expression in liver was analyzed by Western blot. Immunoblots are representative of five independent experiments; GAPDH served as a loading control. Values are expressed as mean ± SEM of five mice. * *p* < 0.05, *** *p* < 0.001 vs. db/db group; ^#^ *p* < 0.05, ^##^ *p* < 0.01 vs. WT group.

The hepatic protein expression levels of SREBP-1, FAS and HMGCR were markedly increased in the obese db/db group compared with that of the WT group. Whereas SREBP-1, FAS and HMGCR protein levels were markedly downregulated by 58.8%, 61.7% and 56.2% in the AS treated group, respectively, as compared with that of the obese db/db group (*p* < 0.05; *n* = 5; Figure 6). These data indicate that AS can suppress TG synthesis in the liver.

### 2.7. Effect of AS on Serum Pro-Inflammatory Cytokines in db/db Mice

**Figure 7:**
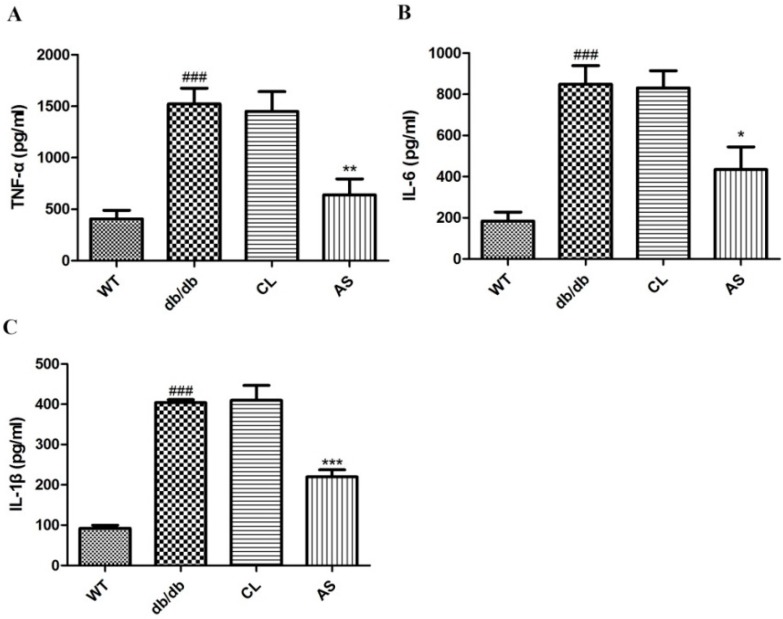
Effect of AS on pro-inflammatory cytokines in db/db mice. Serum (**A**) TNF-α; (**B**) IL-1β and (**C**) IL-6 levels were quantified using enzyme-linked immunosorbent assay (ELISA) kits, following the protocol recommended by the manufacturer. Values are expressed as mean ± SEM of 5~6 mice. * *p* < 0.05, ** *p* < 0.01, *** *p* < 0.001 vs. db/db group; ^###^ *p* < 0.001 vs. the WT group.

As shown in Figure 7, serum TNF-α, IL-6 and IL-1β levels in obese db/db group were increased by 3.75-, 4.61- and 4.39-fold when compared with that of the WT group, whereas administration of AS markedly suppressed the increase in the levels of serum pro-inflammatory cytokines by 49.1%, 41.1% and 45.6%, respectively, when compared with that of the obese db/db group. These results demonstrate that AS exerts the regulation effect of the hepatic inflammation.

## 3. Experimental Section

### 3.1. Reagents and Antibodies

Traditional herbal Zicao were purchased from Xinjiang in China. TNF-α, IL-1β, and IL-6 ELISA Kits and anti-β-actin and anti-glyceraldehyde-3-phosphate dehydrogenase (GAPDH) were purchased from Boster Co. (Wuhan, China). Liver lipid content (A110-1 Triglycerides Assay kit; 100 μL) was purchased from Jiancheng Bioengineering Co. (Nanjing, China). Antibodies against HSL (Ser563), ATGL and horseradish peroxidase (HRP)-conjugated anti-rabbit secondary antibody were purchased from Cell Signaling Technology (Beverley, MA, USA). Antibodies against perilipin, SREBP-1, FAS and HMGCR were obtained from Abcam (Cambridge, UK). All other reagents were purchased from Sigma-Aldrich (St. Louis, MO, USA) unless otherwise specified.

### 3.2. Mice and Experimental Design

Mice experiments were performed in accordance with the Guidelines and were approved by the Animal Research Center of Sun Yat-sen University. Male wild-type C57BLKS mice and C57BLKS/Lepr^db^ (db/db) mice were purchased from the Model Animal Research Center of Nanjing University (MARC, Nanjing, China). Mice (*n* = 24, 6 weeks old) were housed three per cage in controlled environment with a constant temperature of 18 °C–22 °C and a humidity of 55%–60% on a 12:12-h light/dark cycle. After acclimation for seven days, mice were supplied with food and water freely every day. Mice were randomly divided into WT control, obese db/db control, CL, and AS (*n* = 6/group) groups. Mice were immediately administered by oral gavage 100 mg/kg/day CL or 540 mg/kg/day AS, and the WT and obese db/db groups received the same volume of distilled water for eight weeks. The food intake and body weight of the mice were measured weekly during the experimental period. CL is a long acting β-2 adrenergic agonist. In addition, CL, with a recommended dose of 20 to 40 μg orally, is used as a bronchodilator in humans in Europe and Latin America [18]. Therefore, we used CL to compare with AS because its mechanism is very similar to AS in reducing weight losses. In other words, they both reduce body fat and increase basal lipolysis through increasing energy expenditure.

### 3.3. Abdominal Computed Tomography Analysis

Mice were under 4% chloral hydrate in anesthesia and experimented for abdominal computed tomography analysis through micro-computed tomography (micro-CT). Mice were performed with an animal positron emission tomography (PET)/CT/single photon emission computed tomography (SPECT) system (INVEON, Siemens, Knoxville, TN, USA). The computed tomography pictures of mice were analyzed and processed using Siemen’s Inveon software to calculate the volume of the fat mass between lumbar vertebrae one to five.

### 3.4. Liver Lipid Content

The determination of liver lipid content were measured and extracted with CHCl_3_-methanol (1:1) according to the method of Folch et al. [19]. 100 mg of liver tissue was homogenized in 0.2 mL of phosphate buffer (pH 7.4) using an ultrasonic irradiation (SONICS, Newtown, CT, USA). Lipids were extracted by mixing with lysate sample and CHCl_3_-methanol. Subsequently, the homogenates were subjected to assays for lipid content using commercially available kits.

### 3.5. Histological Analysis

For the morphology examination, mice were sacrificed by an intraperitoneal injection of 4% chloral hydrate at the eighth week. The same section of liver and epididymis adipose tissue were fixed with 10% buffered formalin for one day. The tissue was embedded in paraffin after dehydration in ethanol, and cut into sections at a thickness of 5 μm, which were stained with hematoxylin and eosin. In addition, Oil Red-O staining was performed in frozen liver sections to detect the lipid droplets in the hepatocytes. One frozen section of liver tissue was cut into for 5 μm, which were stained with Oil Red-O. Images at 200× magnification were photographed using an IX71 inverted light microscope (Olympus, Tokyo, Japan).

### 3.6. Biochemical Parameters Analysis

At the end of the experiment, mice were anesthetized and sacrificed after an overnight fast. Serum was collected from the abdominal vena cava and separated by centrifugation at 1000 *g* for 10 min at room temperature. Serum TG, FFA, glucose, cholinesterase (CHE), AST, and ALT levels were measured using a Model 7180 automated biochemical analyzer (Hitachi, Tokyo, Japan), according to the manufacturer’s instructions. Inflammatory cytokine levels were quantified using ELISA kits specific for mouse serum TNF-α, IL-1β, and IL-6 levels, following the protocol recommended by the manufacturer.

### 3.7. Western Blot Analysis

Total protein was collected from tissues with lysis buffer containing a protease inhibitor cocktail followed by centrifugation at 12,000 rpm for 15 min 4 °C. Protein concentration was determined using a bicinchoninic acid kit. Equal amounts of proteins were electrophoresed by 6% or 8% sodium dodecyl sulfate polyacrylamide gel electrophoresis and then transferred onto polyvinylidene difluoride membranes (Millipore, Billerica, MA, USA). The membranes were blocked with 5% skim milk powder in tris-buffered saline with Tween 20 (TBST; 0.1% Tween 20, 20 mM Tris-base, 150 mM NaCl, pH 7.5) for 2 h. The membranes were then incubated overnight at 4 °C with primary antibodies against perilipin, ATGL and HSL, SREBP-1, FAS and HMGCR (1:1000). After washing three times with TBST, membranes were incubated with a secondary HRP-conjugated goat anti-rabbit IgG (1:1000) for 1.5 h. β-actin or GAPDH antibody (1:4000) was used as a loading control. Protein bands were visualized using enhanced chemiluminescence (Beyotime, Shanghai, China).

### 3.8. Statistical Analysis

All data were carried out using Graph Pad Prism v.5.0 for Windows (GraphPad, La Jolla, CA, USA). The results are expressed as mean ± SEM. Differences between groups were performed with the one-way analysis of variance (ANOVA), followed by a post hoc comparison with the Bonferroni test. The data were considered statistically significant at *p*-values < 0.05.

## 4. Discussion

Obesity has become an international public health problem that affects people quality of life and is associated with various kinds of diseases. NAFLD undergoes an upward trend with the increased prevalence and incidence of obesity and T2DM [20]. Some anti-obesity drugs have been approved by the U.S. Food and Drug Administration for chronic obesity management. However, most anti-obesity drugs have been limited by their various side effects in the clinic. Orlistat, a lipase inhibitor, reduces weight by 3 kg by inhibiting gastrointestinal lipases; however, it is associated with hepatotoxicity and adverse gastrointestinal effects [21]. Sibutramine, a norepinephrine and serotonin reuptake inhibitor, reduces weight by 4–5 kg on average by suppressing appetite, but it may increase the risk of mania or panic, psychosis, and cardiovascular diseases [22]. CL, a long acting beta-2 adrenergic agonist, has been shown to reduce body fat and increase basal lipolysis through increasing energy expenditure in mice [23]. However, it was accompanied by cardiac diseases and hypokalemia [24]. However, anti-obesity has unique β_3_-adrenergic effects of CL, unrelated to its brochodialating effects, which has helped CL reduce body fat and increase basal lipolysis. Many studies have elaborated that CL increases adipocyte lipolysis, glycolytic capacity, and minimizes protein degradation in animals and humans. We used CL to compare with AS because its mechanism is very similar to AS in reducing weight losses. In other words, they both reduce body fat and increase basal lipolysis through increasing energy expenditure. However AS is an efficient anti-obesity drug from natural sources, unlike drugs above with seriously side effects. Up to now, many studies have demonstrated the anti-tumor, anti-bacterial, anti-viral, anti-inflammatory, and wound healing effects of Zicao [15,25,26]; our study indicates other important pharmacologic effects of Zicao.

In the present study, we investigate the anti-obesity and anti-NAFLD effects of AS in spontaneous obese db/db mice. Eight weeks administration of AS significantly reduces body weight gain, fat tissue, and liver weight without affecting food intake. There is a significant reduction in abdominal fat mass and BMI in the AS group compared with that of the obese db/db group. A study has been reported that the prevalence rate of NAFLD is accompanied with increasing BMI [27]. Both abdominal fat mass and BMI are the increased risk factors for NAFLD, T2DM, and cardiovascular diseases [28]. In addition, our studies also provide evidence that AS inhibits hepatic lipid droplet accumulation. Moreover, serum FFA, TG, AST, and ALT levels, which were elevated in db/db mice, were decreased by AS treatment. In addition, the levels of serum TNF-α, IL-1β and IL-6 are increased in db/db mice compared with that of WT mice, whereas treatment of AS markedly suppressed the increase in the levels of inflammatory cytokines. These results indicate that AS can ameliorate the symptoms of obesity and NAFLD.

Obesity is a complex metabolic syndrome associated with NAFLD [29]. Many studies have found that lipolysis may play important roles in regulating obesity and related metabolic disorders such as fatty liver disease and diabetes mellitus [30]. Adipocyte lipolysis is through increasing in intracellular cyclic AMP levels, which activates the activation of protein kinase A and then enhances the expression of downstream molecules HSL, ATGL and perilipin, as a result of the release of FFA and glycerol from TG [31,32]. We evaluated the effect of AS treatment on the expression of HSL, ATGL and perilipin in adipose tissue by Western blotting, which demonstrated that AS can increase lipolysis by upregulating the expression of perilipin, ATGL and HSL in adipose tissue.

NAFLD develops and progresses to severe liver diseases, coronary atherosclerotic heart disease, and acute pancreatitis [33]. Lipid metabolism contains lipolysis and lipid synthesis. SREBP-1, a gene encoding of hepatic transcription, regulates fatty acid metabolism and lipid synthesis in the liver. In addition, HMGCR, a crucial enzyme of cholesterol synthesis, is involved in fatty acid metabolism and regulates serum FFA level. Furthermore, SREBP-1 also regulates the expression of enzymes (such as acetyl-CoA carboxylase and FAS) involved in mediating hepatic synthesis of fatty acids, TGs and cholesterol [34]. These cascades eventually increase fat accumulation within hepatocytes due to the amount of fatty acids entering the liver. In this study, we showed that the treatment of db/db mice with AS resulted in a decrease of SREBP-1, FAS and HMGCR lipoprotein levels. Collectively, these findings suggest that AS exerts the treatment of NAFLD through regulating hepatic lipid and lipoprotein metabolism.

With the rising incidence of obesity worldwide, NAFLD has become a national public health concern. Moreover the pathogenesis of NAFLD has not been fully elucidated. A ‘two-hit’ hypothesis regarding the complex pathogenetic mechanism of NAFLD/NASH has traditionally been proposed. The ‘first hit’ is associated with reversible deposition of TGs within hepatocytes; however, the ‘second hit’ is in combination with the generation of free radicals, oxidative stress reaction and the production of inflammatory cytokines that progresses eventually to liver fibrosis [35]. NAFLD pathophysiologic processes linking obesity enhanced circulating concentrations of pro-inflammatory cytokines and factors (e.g., TNF-α, IL-6 and IL-1β). TNF-α and IL-1β, pro-inflammatory and lipogenic factors, play crucial roles in the pathogenesis of NAFLD/NASH (liver steatosis, necrosis, apoptosis and fibrosis) [36]. IL-6, another pro-inflammatory cytokine, has been presented to directly and indirectly injure hepatocytes and induce inflammation [37]. The above pro-inflammatory cytokines provoke the production of other cytokines and then recruit inflammatory cells, resulting in liver injury and hepatic fibrosis; however, they are barely found in the serum of healthy mice [38]. Therefore, in our study, TNF-α, IL-6 and IL-1β were marked as representative inflammatory factors involved in the inflammation of liver. We demonstrated that the treatment of db/db mice with AS resulted in a decrease of TNF-α, IL-6 and IL-1 levels. Taken together, AS exerts the effective protection against the development of hepatic steatosis, which may be due to the anti-inflammatory property.

## 5. Conclusions

Obesity and NAFLD are considered important parts of the metabolic syndrome worldwide. The herbal compound of AS is appeared to be an effective therapeutic agent that exerts effective protection against the obesity and NAFLD in a model of spontaneous obese db/db mice by the regulation of lipid metabolism and anti-inflammatory effect. Further studies focusing on the effects of AS on liver fibrosis are required.

## Figures and Tables

**Table 1:** AS reduced the blood lipids and hepatic enzyme levels in db/db mice.

Parameter	WT	db/db	CL	AS
TG (mM)	0.65 ± 0.13	1.92 ± 0.63 ^##^	1.82 ± 0.75	0.83 ± 0.15 **
FFA (mM)	0.58 ± 0.07	1.26 ± 0.19 ^##^	0.76 ± 0.20 **	0.68 ± 0.11 **
Glucose(mM)	5.72 ± 1.01	27.80 ± 4.07 ^##^	25.75 ± 5.96	18.32 ± 2.63 **
CHE(mM)	3.40 ± 0.45	6.89 ± 0.57 ^##^	6.63 ± 0.73	3.76 ± 0.37 **
AST(IU/L)	73.63 ± 8.33	116.42 ± 22.59 ^##^	112.43 ± 23.18	84.78 ± 12.23 *
ALT(IU/L)	20.82 ± 3.15	94.48 ± 24.90 ^##^	99.02 ± 39.22	51.170 ± 14.74 *

TG, triglyceride; FFA, free fatty acid; CHE, cholinesterase; AST, alanine aminotransferase; ALT, alanine aminotransferase. Values are expressed as mean ± SEM of 5~6 mice. * *p* < 0.05, ** *p* < 0.01 vs. db/db group; ^##^ *p* < 0.01 vs. WT group.

## Abbreviations

AS	acetylshikonin
WT	Wild Type
CL	clenbuterol
NAFLD	nonalcoholic fatty liver disease
NASH	nonalcoholic steatohepatitis
TG	triglyceride
FFA	free fatty acid
CHE	cholinesterase
AST	alanine aminotransferase
ALT	alanine aminotransferase
SREBP-1	sterol regulatory element-binding protein-1
FAS	fatty acid synthetase
HMGCR	3-hydroxy-3-methylglutaryl-coenzyme A reductase
HSL	hormone-sensitive lipase
ATGL	adipose TG lipase
GAPDH	glyceraldehyde-3-phosphate dehydrogenase
ELISA	enzyme-linked immunosorbent assay
TNF-α	tumour necrosis factor-a
IL-6	interleukin-6
IL-1β	interleukin-1β BMI body mass index
T2DM	type 2 diabetes mellitus
ANOVA	one-way analysis of variance
